# Socioeconomic inequalities in the healthiness of food choices: Exploring the contributions of food expenditures

**DOI:** 10.1016/j.ypmed.2016.04.012

**Published:** 2016-07

**Authors:** Rachel Pechey, Pablo Monsivais

**Affiliations:** a Behaviour and Health Research Unit, Institute of Public Health, University of Cambridge, Cambridge CB2 0SR, UK; b Centre for Diet and Activity Research, University of Cambridge, School of Clinical Medicine, Box 285, Institute of Metabolic Science, Cambridge CB2 0QQ, UK

**Keywords:** Socioeconomic factors, Food and beverages, Health behavior, Consumer behavior

## Abstract

Investigations of the contribution of food costs to socioeconomic inequalities in diet quality may have been limited by the use of estimated (vs. actual) food expenditures, not accounting for where individuals shop, and possible reverse mediation between food expenditures and healthiness of food choices. This study aimed to explore the extent to which food expenditure mediates socioeconomic inequalities in the healthiness of household food choices. Observational panel data on take-home food and beverage purchases, including expenditure, throughout 2010 were obtained for 24,879 UK households stratified by occupational social class. Purchases of (1) fruit and vegetables and (2) less-healthy foods/beverages indicated healthiness of choices. Supermarket choice was determined by whether households ever visited market-defined high-price and/or low-price supermarkets. Results showed that higher occupational social class was significantly associated with greater food expenditure, which was in turn associated with healthier purchasing. In mediation analyses, 63% of the socioeconomic differences in choices of less-healthy foods/beverages were mediated by expenditure, and 36% for fruit and vegetables, but these figures were reduced to 53% and 31% respectively when controlling for supermarket choice. However, reverse mediation analyses were also significant, suggesting that 10% of socioeconomic inequalities in expenditure were mediated by healthiness of choices. Findings suggest that lower food expenditure is likely to be a key contributor to less-healthy food choices among lower socioeconomic groups. However, the potential influence of cost may have been overestimated previously if studies did not account for supermarket choice or explore possible reverse mediation between expenditure and healthiness of choices.

## Highlights

• Large dataset linking detailed food expenditure data with healthiness of choices.
• Lower food expenditure mediates less-healthy choices for lower SES groups.
• Reverse mediation was also observed, however; albeit to a smaller degree.
• The potential influence of cost may have been overestimated previously.

## 1 Introduction

A body of evidence shows that purchasing and consumption of unhealthy diets, in particular, eating fewer fruits and vegetables, is strongly patterned by socioeconomic status (SES) (Appelhans et al., 2012, Darmon and Drewnowski, 2008, Giskes et al., 2010, Pechey et al., 2013, UK Department for Environment, Food and Rural Affairs, 2011). One likely contributor to the socioeconomic patterning in healthy diets is the cost of food: less nutritious, energy-dense foods are often cheaper sources of calories (Drewnowski, 2010, Jones et al., 2014), and higher diet quality has been associated with higher diet cost (Bernstein et al., 2010, Lee et al., 2011, Rao et al., 2013, Rehm et al., 2015). Moreover, given most research to date has estimated diet cost by linking dietary intake data to prevailing food price data, the socioeconomic inequalities in expenditure may have been underestimated by assuming a constant price for particular foods (i.e. only accounting for differences between types of foods purchased and not variation between brands) (Monsivais et al., 2013). Even so, dietary cost explains some of the relationship between SES and nutrient density of consumed foods (Monsivais et al., 2010), and estimated diet cost has also been shown to mediate the pathway between socioeconomic status (income) and diet quality in a US sample (Aggarwal et al., 2011).

A potentially related avenue of research has suggested that consumers who patronize low-priced supermarkets are more likely to have lower-quality diets (Aggarwal et al., 2014b) and higher BMI (Chaix et al., 2012, Drewnowski et al., 2012, Lear et al., 2013). Yet even within the same store, more educated households have been found to make healthier purchases (Handbury et al., 2015). One contributing factor may be the prioritization of low cost, which may lead to preferences for certain supermarkets and also limit food choices within store (Aggarwal et al., 2014a, Pechey and Monsivais, 2015). As such, concerns about cost may be driving some of the association between supermarket price tier and healthiness of diet. Conversely, if individuals choose to patronize a particular supermarket for reasons other than price (for example, believing a store to offer a wider range of healthy products), this choice may still contribute to subsequent diet cost, given expenditure is a consequence of customers' product choices. Both choice of products within store and choice of the store itself are likely to depend in part on individuals' motivations (e.g., price, health, convenience), which may vary by SES (Pechey et al., 2015). As such, the relationship between diet cost and diet quality may be bi-directional. Although the extent of this bi-directionality cannot be determined in cross-sectional analyses, reverse mediation analyses offer an initial exploration of the potential contributions of each factor to socioeconomic inequalities.

Of particular interest is the extent to which financial motivations mediate socioeconomic differences in healthiness of choices, given that this would likely indicate the potential effects of changing food prices on healthiness of diet. However, financial motivations are often not possible to reliably measure directly and instead food expenditure has often been used as a proxy. Food expenditure may be influenced by supermarket choice, which may in turn have been influenced by financial motivations, but may also have been determined by other motivations (e.g. convenience). As such, in the current study we will investigate whether food expenditure mediates the pathway between socioeconomic status and healthiness of choices, with and without controlling for supermarket choice. By supermarket choice, we potentially control for the likely self-selection to a given type of supermarket. On the other hand, without controlling for supermarket choice, we run the risk of ignoring that selection of supermarket is not necessarily a free choice (e.g. it may be limited by geographic access, which could be associated with SES). So the above mediation models may reflect upper and lower bounds. As such, this study aims to explore a range of values for the possible mediation of expenditure on the socioeconomic differences in healthiness of choices.

This study extends explorations of the role of food costs as a mediator of socioeconomic inequalities in healthiness of choices; firstly, by looking at actual expenditure (rather than estimated diet costs) in a large UK sample. Secondly, reverse mediation between food expenditure and healthiness of choices will be explored. Thirdly, it will explore the associations between SES, food expenditure and healthiness of choices alongside the contribution of supermarket choice.

## 2 Methods

### 2.1 Sample

Data were obtained from the Kantar WorldPanel (KWP) UK household survey from 2010 (as this involved analyzing de-identified existing data, ethical approval was not required). The sample consists of an ongoing panel, originally recruited via post or email to be representative of the UK in terms of age group, household size and region of residence. Households must meet minimum volume and spending criteria based on household size for inclusion, based on 4-week purchasing blocks. Further details of sample recruitment and quality control have been described elsewhere (Pechey and Monsivais, 2015).

Participating households (n = 24,879) recorded all food and beverage purchases brought home (i.e. excluding purchases that were consumed away from home), including volume purchased, spend, nutritional content, and the retail chain from which products were purchased. Sociodemographic data including number of adults and children in each household, ages and genders of household members, and socioeconomic indicators were also collected.

### 2.2 Measures

#### 2.2.1 Socioeconomic status

Head-of-household occupation using the UK Registrar General's social class classification (Rose and Pevalin, 2001) was categorized into three groups: Higher Managerial and Professional (‘Higher’: n = 5332); White Collar and Skilled Manual (‘Middle’: n = 13,621); and Semi-skilled and Unskilled Manual (‘Lower’: n = 5926).

#### 2.2.2 Food expenditure

Expenditure was calculated from the households' total spend (£) on take-home food and beverages over the 52 week period, divided by the total number of calories those purchases for the same period, multiplied by 2000 to give an energy-adjusted food expenditure variable (£ per 2000 kcal).

#### 2.2.3 Supermarket choice

We defined supermarket choice as in Pechey and Monsivais (2015): firstly, supermarkets were categorized as high-, medium- or low-cost based on market definitions (Food and Drink Economics branch: DEFRA, 2006, USDA Foreign Agricultural Service: Global Agriculture Infor). Households were then classified according whether or not they ever patronized high- or low-cost supermarkets (in addition to medium-cost supermarkets, which were almost universally patronized), giving four groups: Used low-cost supermarkets exclusively or low- and medium-cost supermarkets (‘Low-cost’); Used medium-cost supermarkets only (‘Medium-cost’); Used high-cost supermarkets exclusively or medium- and high-cost supermarkets (‘High-cost’); Used all three tiers of supermarkets (‘All-types’).

#### 2.2.4 Healthiness of food and beverage choices

Two outcome variables assessed healthiness of food and beverage choices, comprising less-healthy and healthier indices:

1. Percentage of food energy purchased from less-healthy foods and non-alcoholic beverages, as classified by FSA Nutrient Profile (Rayner et al., 2005) scores for individual products (Scores are calculated from the energy, saturated fat, sugar, sodium, fiber, protein, and fruit, vegetable and nut content, per 100 g; foods scoring 4 or more, and beverages 1 or more, are categorized as less-healthy).
2. Percentage of food energy purchased from fruit and vegetables — this included fresh, canned, frozen and dried fruit, vegetables and legumes, but excluded juice, potatoes, and fruit and vegetables present in processed products.

### 2.3 Statistical analysis

**Fig. 1:**
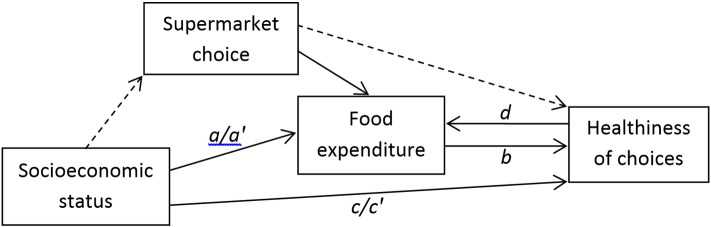
Possible pathways linking socioeconomic status with healthiness of food choices. Dashed lines represent pathways that have been reported elsewhere; solid lines indicate relationships to be explored in this paper: a1: Pathway from socioeconomic status to food expenditure; a2: Pathway from socioeconomic status to food expenditure, controlling for supermarket choice; b: Pathway from food expenditure to healthiness of choices; c: Pathway from socioeconomic status to healthiness of choices; c′: Pathway from socioeconomic status to healthiness of choices, controlling for food expenditure; d: Pathway from healthiness of choices to food expenditure.

Firstly, multiple regression analyses were conducted to explore the pathways linking ‘Socioeconomic status’ → ‘Food expenditure’ → ‘Healthiness of choices’ in this dataset, estimating:

1. Food expenditure by SES (using dummy variables) (*pathway* ‘*a1*/*a2*’ *in* Fig. 1)
2. The percentage of energy purchased from (a) less-healthy foods/beverages and (b) fruit and vegetables by:
  i. expenditure (*pathway* ‘*b*’); and/or
  ii. SES (*pathway* ‘*c*’)

Mediation analyses (conducted using the product of coefficients method with bootstrapped standard errors) then examined the role of expenditure as a potential mediator of socioeconomic inequalities in healthiness of choices (i.e. ‘*Socioeconomic status*’ → ‘*Food expenditure*’ → ‘*Healthiness of choices*’: *pathways* ‘*a1*/*a2*’, ‘*b*’ *and* ‘*c′*’ *in* Fig. 1), as well as the reverse pathway (the role of healthiness of choices as a potential mediator of socioeconomic inequalities in expenditure, i.e. ‘*Socioeconomic status*’ → ‘*Healthiness of choices*’ → ‘*Food expenditure*’ *in* Fig. 1 (‘*a1*/*a2*’, ‘*c′*’ *and* ‘*d*’)). Socioeconomic status was indicated by three ordinal levels of occupational social class, modelled using dummy variables, running separate analyses for Higher vs. Middle occupational social class and Higher vs. Lower occupational social class (with Higher occupational social class as the reference group in both analyses). These estimates were then aggregated to give the total indirect and direct effects of expenditure as a mediator of occupational social class in healthiness of choices. Model estimates reflect the difference in purchase of each food group associated with decreasing occupational social class. To examine the impact of supermarket choice on these relationships, analyses were conducted with and without controlling for supermarket choice (i.e. *comparing* ‘*a1*’ *and* ‘*a2*’).

Analyses (using Stata MP version 13 (StataCorp, College Station, USA)) used robust standard errors, given evidence of heteroscedasticity, and the percentage of energy from fruit and vegetables and expenditure were log-transformed to address positively skewed distributions. Reported significance levels were adjusted for multiple testing using Bonferroni's correction. Analyses are reported in terms of a 20% increase in expenditure, which equates to an approximately £0.65 increase in spend per 2000 cal at the median value of expenditure (£3.24 per 2000 cal), and would move a household at the median value within each expenditure quintile into the quintile above.

Regressions controlled for a number of potential confounders including age, gender, and ethnic group (white/non-white) of main shopper; number of adults in household, number of children in household, and for region of residence (Midlands, North East, Yorkshire, Lancashire, South, Scotland, Anglia, Wales & West, South West and London). Sensitivity analyses using hierarchical models by region, to account for possible clustering effects, produced very similar results, which we present in the Supplementary Materials.

## 3 Results

### 3.1 Descriptive analyses

The sample characteristics, stratified by quintile of food expinditure, are presented in Table 1. Higher food expenditure was associated with higher mean age of the main food shopper and fewer children in the household. Highest-spending households were also most likely to identify as white and reside in the London region. Table 1 also revealed socioeconomic inequalities in expenditure, with those from lower occupational social classes and with lower incomes tending to fall into lower quintiles of expenditure. Supermarket choice appeared to be associated with expenditure, with 59% of those in the lowest quintile of expenditure shopping at low-/medium-cost stores compared to 16% in the highest expenditure quintile. Conversely, 3% of the lowest expenditure quintile vs. 29% of the highest quintile shopped at high-/medium-cost stores.

Table 1 also suggested a trend with those in the higher quintiles of expenditure purchasing higher percent energy from fruit and vegetables and lower percent energy from less-healthy foods and beverages than those in lower quintiles of expenditure.

### 3.2 Multiple regression analyses

#### 3.2.1 Expenditure

**Fig. 2:**
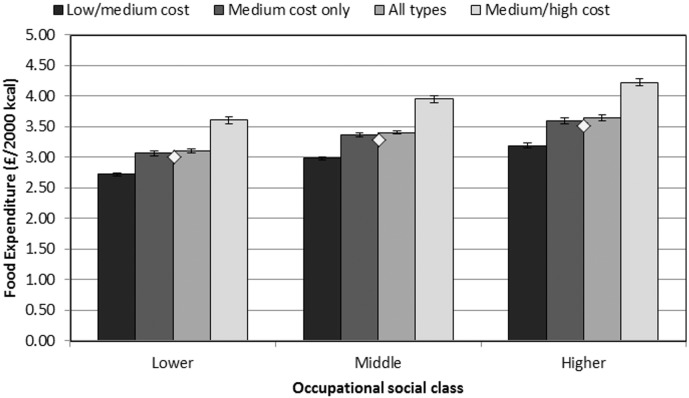
Adjusted means: Socioeconomic inequalities in expenditure by supermarket choice group. Error bars show Bonferroni-adjusted 95% CIs; diamonds show main effect of occupational social class without controlling for supermarket choice (all significantly different at p < 0.05). Data from UK, 2010. Regressions controlled for age, gender and ethnic group of main shopper; number of adults in household, number of children in household, and region of residence. Coefficients have been back-transformed (by exponentiating the B coefficients) as expenditure was log-transformed in analyses. Analyses used robust standard errors. For occupational social class, Higher: Higher Managerial and Professional; Middle: White Collar and Skilled Manual; Lower: Semi-skilled and Unskilled Manual.

Fig. 2 shows the results of a regression analysis estimating expenditure among occupational social class and supermarket choice groups, with both higher SES and higher-cost supermarkets being significantly associated with greater expenditure. The variation in expenditure between supermarket choice groups (approximately £0.90–£1 difference between groups) was greater than the variation between SES groups (around £0.50–£0.60). This variation by supermarket choice groups was consistent across SES groups, with heterogeneity in food expenditure even in the lower SES group.

#### 3.2.2 Healthiness of choices

Table 2 shows the results of the three models of expenditure as a predictor of the healthiness of choices. In terms of expenditure, in the basic model (Model 1: expenditure and demographic variables only) a 20% increase (equating to a household at the median value within each expenditure quintile moving into the quintile above) was associated with a 0.7 percentage point decrease in the percent energy from less-healthy foods and beverages, whereas a 20% increase in expenditure was associated with a 7.2% increase in percent energy from fruit and vegetables. There was little change to the coefficients for expenditure between the different models (when adding occupational social class (Model 2) and then supermarket choice (Model 3)), although the increase in percent energy from fruit and vegetables was slightly reduced, to 6.4%.

### 3.3 Mediation analyses

**Fig. 3:**
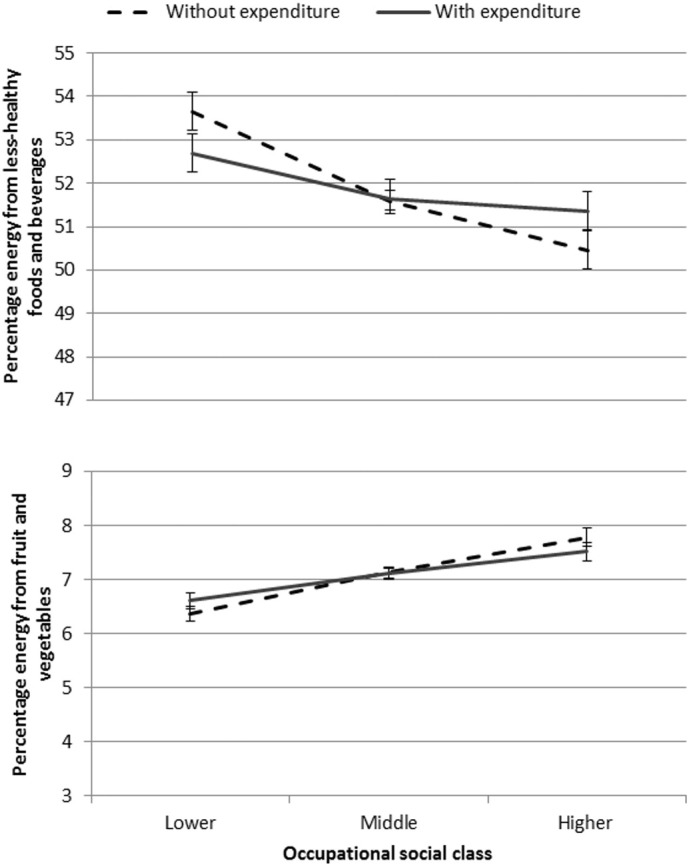
Adjusted means: Socioeconomic inequalities in healthiness of choices, with and without controlling for expenditure. Error bars show Bonferroni-adjusted 95% CIs. Data from UK, 2010. Regressions controlled for age, gender and ethnic group of main shopper; number of adults in household, number of children in household, and region of residence. Coefficients have been back-transformed (by exponentiating the B coefficients) as expenditure was log-transformed in analyses. Analyses used robust standard errors.

Fig. 3 shows socioeconomic inequalities in healthiness of choices, with and without controlling for expenditure, suggesting that socioeconomic inequalities in healthiness of choices was reduced when expenditure was included in the models for both outcomes.

Table 3 shows the results of mediation analyses examining whether expenditure mediates the relationship between SES and healthiness of choices, with and without controlling for supermarket choice. For purchase of less-healthy foods and beverages 63% of the association was mediated without controlling for supermarket choice, whilst the equivalent figure for purchase of fruit and vegetables was 36%.

For both outcomes, controlling for supermarket choice reduced the indirect effect (and the proportion mediated: to 53% and 31% respectively).

The reverse mediation analyses were also conducted to examine whether healthiness of choices mediates the pathway between SES and expenditure. For percent energy from less-healthy foods and beverages, 11% of the total effect was mediated (indirect effect: − 0.03; Bonferroni-corrected 95% CIs: − 0.04, − 0.02) without controlling for supermarket choice group, and 13% when controlling for supermarket choice. Similar results were obtained for percent energy from fruit and vegetables (without supermarket choice group: 11%; indirect effect: − 0.03; Bonferroni-corrected 95% CIs: − 0.04, − 0.03; with supermarket choice group: 12%).

Analyses from hierarchical regression models that clustered households within regions (rather than adjusting for region) showed similar results. Supplementary Table S1 shows percent energy purchased from less-healthy foods and from fruits and vegetables estimated from multivariable regression models (like Table 2) and again from hierarchical models. Additionally, estimated food expenditure across socioeconomic groups was similar whether based on hierarchical models or models that adjusted for region (Supplementary Table S2).

## 4 Discussion

This analysis of a large UK dataset, employing detailed scanner data, suggests that food costs may be an important contributor to socioeconomic inequalities in healthiness of food and beverage choices. Our analyses (using actual rather than estimated food expenditure) supported the findings of previous studies in that higher SES households were found to have significantly higher food spending and also had somewhat healthier patterns of food purchasing (Appelhans et al., 2012, Giskes et al., 2010, Pechey et al., 2013, Darmon and Drewnowski, 2008). Independent of SES, food expenditure had a small positive association with healthier patterns of food and beverage purchasing (Appelhans et al., 2012). In order to explore these relationships in more detail, we investigated the role of expenditure as a mediator of socioeconomic inequalities in healthiness of choices, going beyond previous research by analysing: (1) reverse mediation and (2) the likely range of values for these mediation effects in this dataset by comparing analyses with and without controlling for supermarket choice.

### 4.1 Expenditure as a mediator

Further exploration of the role of expenditure suggested this may be a significant mediator of socioeconomic inequalities in healthiness of food and beverage choices, as has been found in US studies, using different indicators of SES and diet quality (Aggarwal et al., 2011). In addition, while the association between expenditure and healthiness of choices was larger for fruit and vegetables than for less-healthy items, the extent to which expenditure mediated socioeconomic inequalities was greater for less-healthy foods and beverages (63%) than for fruit and vegetables (35%). This may tie in with previous findings that less mediation was seen when looking at the mean adequacy ratio (representing micronutrients in the diet, and perhaps healthier purchases) than when using energy density, which is likely to reflect the ratio of healthier to less-healthy foods and beverages (Aggarwal et al., 2011).

### 4.2 Healthiness of choices as a mediator

However, the reverse mediation pathway (healthiness of choices as a mediator of socioeconomic inequalities in expenditure) was also significant, albeit appearing smaller. This highlights the need for caution when interpreting mediation results from cross-sectional analyses. The prioritisation of either price or health could lead to socioeconomic patterning of both food expenditure and healthiness of food choices: prioritising price may constrain healthiness of choices, while prioritising health may necessitate higher expenditure. Moreover, prioritisation of price vs. health may vary by socioeconomic group, with previous studies suggesting that lower SES groups are more likely than higher SES groups to prioritise price, and vice versa for prioritising health (Bowman, 2006, Konttinen et al., 2013, Pechey et al., 2015). One question of interest to public health researchers is whether changing food costs could influence healthiness of choices and/or on socioeconomic inequalities in choices. If the reverse mediation does indicate a bi-directional relationship, one consequence may be that the possible range of effects of price changes on socioeconomic differences in diet healthiness are prone to over-estimation in analyses where causality is not determined.

### 4.3 Supermarket choice and expenditure

Supermarket choice was systematically associated with food expenditure (see Table 1). To explore the role of supermarket choice in these associations between SES, food expenditures and healthiness of choices, we additionally controlled for this variable in the analyses, finding this had no or limited effects on the coefficients for expenditure on healthiness of choices. When controlling for supermarket choice in mediation analyses, the proportion of socioeconomic inequalities in healthiness of choices that was mediated by expenditure was reduced for both of the outcome variables, though even controlling for supermarket tier, we observed substantial mediation of socioeconomic inequalities by expenditure. Individuals' choice of supermarket may be determined in part by factors directly linked to expenditure, e.g. financial constraints, but are also likely to take account of other motivations (such as convenience, perceptions of quality or preferences for particular product ranges). Supermarket choice may influence expenditure for patrons to some extent regardless of their initial motivations. By comparing the mediation effects with and without supermarket choice, this study allows us to examine a likely range of values for the mediation effects of food expenditure in this dataset. As such, the influence of supermarket choice in these mediation analyses may in part represent other motivations that may influence healthiness of food choices and vary by SES, for example, health attitudes or knowledge (Aggarwal et al., 2014b, McKinnon et al., 2014, Turrell and Kavanagh, 2006).

### 4.4 Implications for research

By exploring the role of expenditure across these different analyses, this study considers the potential contribution of food costs to socioeconomic differences in the healthiness of food choices. While each of the analyses in this study suggested that food costs were likely to be playing a role in socioeconomic differences in food purchasing choices, this paper highlights uncertainties in determining the size of this contribution. In order to explore this more fully, future studies investigating the pathways illustrated in Fig. 1, and in particular, integrating households' motivations in choosing stores and products, would be beneficial. Such analyses might help establish the extent to which these different pathways may reflect different households' behaviour (including the extent to which supermarket choice may result from financial constraints or other motivations), and to what extent this varies by SES. In particular, integrating these results alongside those of experimental or intervention studies could help to disentangle possible bi-directionality in the pathways between expenditure and healthiness of choices. Indeed, the findings are consistent with intervention studies that have provided financial incentives for improving diet; for example, cash-back and subsidy schemes are providing evidence of positive effects of changing food prices on healthiness of choices in low- and middle-income households (An et al., 2013, Klerman et al., 2014, Ni Mhurchu et al., 2010, Waterlander et al., 2012). Unpicking the pathway between socioeconomic status, expenditure and healthiness of food choices in detail could help inform more effective programs and policies to promote healthier food choices.

### 4.5 Methodological considerations and limitations

Several limitations need to be borne in mind, however; not least that the data were cross-sectional. Moreover, these results reflect purchasing, and as such may not translate directly to diet. It should be noted that the overall low volumes of food and beverages recorded in this dataset suggest underreporting (with households reporting on average approximately three quarters of the in-home calories, excluding alcohol, reported in a representative household survey of food spending from the UK in 2010) (Pechey and Monsivais, 2015, UK Department for Environment, Food and Rural Affairs, 2012). However, the underreporting does not seem to vary systematically by SES (Pechey and Monsivais, 2015, UK Department for Environment, Food and Rural Affairs, 2011, UK Department for Environment, Food and Rural Affairs, 2012). In addition, the mean expenditure per calorie is very similar between these two datasets (Living Costs and Food Survey: 0.172 pence/kcal; study dataset: 0.171 pence/kcal).

## 5 Conclusions

In summary, this study involved actual food expenditure data and allowed a more nuanced exploration of the potential role of this variable in the socioeconomic inequalities of healthiness of food choices than has previously been reported. These findings suggest cost is still likely to be a significant contributor to healthiness of food choices, and that a sizeable proportion of the socioeconomic inequalities in healthiness of choices may be mediated by expenditure in a large UK sample. This suggests actual and/or perceived cost of healthy diets may be key factors in tackling socioeconomic disparities in food purchasing choices.

## Conflicts of interest

The authors declare that there are no conflicts of interest.

## Transparency document

Transparency document. Image 1

## Figures and Tables

**Table 1:** Household and main shopper characteristics by expenditure quintiles. Data from UK, 2010.

	Quintile 1	Quintile 2	Quintile 3	Quintile 4	Quintile 5	Total
£/2000 kcal	0.82–2.58	2.58–3.03	3.03–3.48	3.48–4.14	4.14–15.17	
n	4975	4976	4976	4976	4976	24,879
*Household composition (mean (s.d.))*						
Number of adults	2.2 (1.0)	2.1 (0.9)	2.1 (0.8)	2.0 (0.8)	1.9 (0.8)	2.1 (0.9)
Number of children	0.9 (1.2)	0.8 (1.1)	0.7 (1.0)	0.5 (0.9)	0.3 (0.6)	0.6 (1.0)

*Age of main shopper*						
(mean (s.d.))	48.4 (15.5)	48.2 (15.5)	48.7 (15.6)	50.4 (15.7)	52.1 (15.2)	49.5 (15.6)

*Woman is*						
Main shopper (%)	76.1	80.6	81.6	79.7	73.8	78.4

*Ethnic group (%)*						
White (main shopper)	86.7	93.0	93.6	94.2	94.6	92.4
Not available	3.7	3.5	2.7	2.7	2.7	3.1

*Region (%)*						
London	15.2	14.3	16.0	17.9	24.1	17.5
Midlands	18.0	17.6	16.7	16.0	15.3	16.7
North East	5.1	5.9	5.0	5.3	5.0	5.3
Yorkshire	12.0	10.7	11.2	9.4	9.2	10.5
Lancashire	11.5	11.5	12.5	13.0	11.2	11.9
South	10.3	9.9	10.3	9.4	9.7	9.9
Scotland	6.8	8.4	8.8	10.2	8.8	8.6
Anglia	8.8	8.7	8.2	8.2	7.2	8.2
Wales and West	8.7	9.0	7.9	7.5	6.8	8.0
South West	3.7	4.0	3.4	3.2	2.8	3.4

*Occupational social class*^a^*(%)*						
Lower	37.4	28.1	22.3	18.2	13.0	23.8
Middle	50.4	55.4	57.4	56.6	54.0	54.7
Higher	12.2	16.4	20.3	25.2	33.0	21.4

*Equivalised income bands (%)*						
£0–£9999 per annum (pa)	31.6	21.2	15.1	11.4	7.5	17.3
£10,000–£19,999 pa	34.7	38.0	37.6	34.2	27.0	34.3
£20,000–£29,999 pa	6.9	11.3	14.2	16.0	16.8	13.0
£30,000–£39,999 pa	2.5	3.9	7.4	10.4	16.9	8.2
£40,000 + pa	0.8	0.8	1.9	4.0	8.5	3.2
Refused/did not know	23.5	24.7	24.0	24.0	23.4	23.9

*Supermarket choice group (%)*						
Low/medium cost	58.7	46.4	35.7	27.5	15.8	36.8
Medium cost only	16.9	21.4	22.1	22.0	20.3	20.6
All types	21.4	26.4	32.1	34.4	34.7	29.8
High/medium cost	3.0	5.8	10.1	16.1	29.1	12.8

*Healthiness of choices (mean (s.d.))*						
Percent energy from fruit and vegetables^b^	5.7 (3.4)	6.4 (3.5)	6.9 (3.8)	7.5 (4.0)	8.4 (5.1)	7.0 (4.1)
Percent energy from less-healthy foods and beverages^c^	55.6 (10.1)	53.4 (9.0)	51.8 (8.9)	50.4 (8.9)	48.0 (9.8)	51.8 (9.7)

*Purchasin*g* behaviour (mean (s.d.))*						
Total expenditure (£) per person per day	1.54 (1.03)	1.87 (1.00)	2.10 (1.14)	2.43 (1.23)	3.14 (1.68)	2.22 (1.35)
Total calories purchased per person per day	1396 (922)	1332 (708)	1295 (698)	1288 (647)	1245 (635)	1311 (731)

a Occupational social class: ‘Higher’: Higher Managerial and Professional; ‘Middle’: White Collar and Skilled Manual; ‘Lower’: Semi-skilled and Unskilled Manual.

b Fruit and vegetables included fresh, canned, frozen and dried fruit, vegetables and legumes, but excluded juice, potatoes, and fruit and vegetables present in processed products.

c Less-healthy foods and beverages were defined by FSA Nutrient Profile (28) scores for individual products (foods scoring 4 or more, and beverages 1 or more).

**Table 2:** Expenditure (£/2000 kcal)^a^ as a predictor of healthiness of household food choices.

		Percent energy from less-healthy foods and beverages	Percent energy from fruit and vegetables^b^
Model 1: Expenditure (+ control variables)	For 20% increase in expenditure^c^ Coefficient: B (95% CIs)	0.7 percentage point decrease^d^ − 9.32⁎⁎⁎ (− 10.10, − 8.54)	7.2% increase^d^ 0.38⁎⁎⁎ (0.34, 0.42)
Model 2: Model 1 + social class	For 20% increase in expenditure Coefficient: B (95% CIs)	0.7 percentage point decrease − 8.92⁎⁎⁎ (− 9.73, − 8.11)	6.4% increase 0.34⁎⁎⁎ (0.30, 0.38)
Model 3: Model 2 + supermarket choice group	For 20% increase in expenditure Coefficient: B (95% CIs)	0.7 percentage point decrease − 9.45⁎⁎⁎(− 10.31, − 8.59)	6.4% increase 0.34 ⁎⁎⁎ (0.30, 0.38)

All CI estimates were Bonferroni-corrected for multiple comparisons. Regressions controlled for age gender and ethnic group of main shopper; number of adults in household, number of children in household, and region of residence. Less-healthy foods and beverages were defined by FSA Nutrient Profile (28) scores for individual products (foods scoring 4 or more, and beverages 1 or more).

a Expenditure was logged in analyses.

b Percent energy from fruit and vegetables was logged in analyses.

c A 20% increase in expenditure equates to an approximately £0.65 increase in spend per 2000 cal at the median value of expenditure (£3.24 per 2000 cal), and would move a household at the median value within each expenditure quintile into the quintile above.

d Back-transformed from logged variables in analyses, from coefficient B: – For less-healthy foods and beverages, calculated as: B*log(1.2) – For fruit and vegetables, calculated as: 1.2^B.

⁎⁎⁎ p < 0.001.

**Table 3:** Mediation analyses: Expenditure as mediator of socioeconomic inequalities in healthiness of choices. Estimates of indirect and direct effects represent the differences in the purchase of each food category associated with decreasing occupational social class.

		Percent energy from less-healthy foods/beverages	Percent energy from fruit and vegetables^a^
Without supermarket choice group	Indirect effect^b^ (a × b)	2.71⁎(2.32, 3.07)	− 0.10⁎(− 0.12, − 0.09)
Direct effect (c*′*)	1.61⁎(0.60, 2.61)	− 0.18⁎(− 0.24, − 0.13)
Proportion of total effect mediated	63%	36%
With supermarket choice group	Indirect effect^b^ (a′ × b)	2.14⁎(1.81, 2.49)	− 0.08⁎(− 0.09, − 0.06)
Direct effect (c*′*)	1.87⁎(0.86, 2.88)	− 0.18⁎(− 0.24, − 0.13)
Proportion of total effect mediated	53%	31%

Bootstrapped standard errors; bias-corrected confidence intervals in parentheses.

Occupational social class was used as an indicator of socioeconomic status, and modelled using dummy variables, running separate analyses for Higher vs. Middle occupational social class and Higher vs. Lower occupational social class (with Higher occupational social class as the reference group in both analyses). These estimates were then aggregated to give the total indirect and direct effects of expenditure as a mediator of occupational social class in healthiness of choices. Regressions controlled for age, gender and ethnic group of main shopper; number of adults in household, number of children in household, and region of residence. Less-healthy foods and beverages were defined by FSA Nutrient Profile (28) scores for individual products (foods scoring 4 or more, and beverages 1 or more).

a Percent energy from fruit and vegetable and expenditure were log-transformed in analyses.

b See Fig. 1 for pathways a, a′, b and c′.

⁎ p < 0.05.

## Appendix A Supplementary data

Supplementary tables. Image 2
