# Supplementary material for: Socioeconomic inequalities in the healthiness of food choices: Exploring the contributions of food expenditures
Source: Prev Med. 2016 Jul;88:203–9. doi: 10.1016/j.ypmed.2016.04.012 (PMC4910945; doi:10.1016/j.ypmed.2016.04.012)
Supplement: Supplementary file 2 — Supplementary tables. [file mmc2.docx]

| **Supplementary Table S1.** Expenditure (£/2000 kcal) as a predictor of healthiness of household food choices, estimated from multivariable regression models adjusting for region and hierarchical multivariable regression models that clustered households in regions. Data from UK, 2010. | | | | | |
| --- | --- | --- | --- | --- | --- |
|  |  | **Original models^1^** | | **Hierarchical models^2^** | |
|  |  | **Percent energy from less-healthy foods and beverages** | **Percent energy from fruit and vegetables^3^** | **Percent energy from less-healthy foods and beverages** | **Percent energy from fruit and vegetables^3^** |
| **Model 1:** Expenditure (+ control variables) | *Coefficient: B*  *(95% CIs)* | -9.32 ***  (-10.10, -8.54) | 0.38 ***  (0.34, 0.42) | -9.32 ***  (-10.14, -8.50) | 0.38 ***  (0.32, 0.43) |
| **Model 2:** Model 1 + social class | *Coefficient: B*  *(95% CIs)* | -8.92 ***  (-9.73, -8.11) | 0.34 ***  (0.30, 0.38) | -8.92 ***  (-9.77, -8.06) | 0.34 ***  (0.29, 0.39) |
| **Model 3:** Model 2 + supermarket choice group | *Coefficient: B*  *(95% CIs)* | -9.45 ***  (-10.31, -8.59) | 0.34 ***  (0.30, 0.38) | -9.45 ***  (-10.29, -8.62) | 0.34 ***  (0.29, 0.39) |
| **p<0.05; ** p<0.01; *** p<0.001.* **^1^** Original models are those multivariable regression models presented in the main text, which adjust for region (10 regions of the United Kingdom) using dummy variable coding as a means of controlling for possible confounding by region. **^2^** Hierarchical models cluster 24,879 households into 10 regions as a means of accounting for possible clustering effects. **^3^** Percent energy from fruit and vegetables and expenditure were logged in analyses | | | | | |

| **Supplementary Table S2.** Occupational social class and supermarket choice group as predictors of expenditure (£/2000 kcal), estimated from multivariable regression models that controlled for region and hierarchical multivariable regression models that clustered households in regions. Estimates from original models are the same as those displayed in Figure 2. Expenditure was logged for analysis. Data from UK, 2010. | | | | |
| --- | --- | --- | --- | --- |
|  |  |  | **Original**  **models^1^** | **Hierarchical models^2^** |
| **Model 1: Occupational social class** | | | | |
| **Occupational social class** | *Lower* | *Reference group* | --- | --- |
|  | *Middle* | *Coefficient: B*  *(95% CIs)* | -0.09 ***  (-0.11, -0.08) | -0.09 ***  (-0.11, -0.08) |
|  | *Higher* | *Coefficient: B*  *(95% CIs)* | -0.21 ***  (-0.23, -0.19) | -0.21 ***  (-0.23, -0.19) |
|  | | | | |
| **Model 2: Model 1 + supermarket choice group** | | | | |
| **Occupational social class** | *Lower* | *Reference group* | --- | --- |
|  | *Middle* | *Coefficient: B*  *(95% CIs)* | -0.07 ***  (-0.08, -0.05) | -0.07 ***  (-0.08, -0.06) |
|  | *Higher* | *Coefficient: B*  *(95% CIs)* | -0.16 ***  (-0.18, -0.14) | -0.16 ***  (-0.18, -0.14) |
|  |  |  |  |  |
| **Supermarket choice group** | *Low/medium cost* | *Reference group* | --- | --- |
|  | *Medium cost only* | *Coefficient: B*  *(95% CIs)* | 0.12 ***  (0.10, 0.14) | 0.12 ***  (0.09, 0.15) |
|  | *All types* | *Coefficient: B*  *(95% CIs)* | 0.13 ***  (0.12, 0.15) | 0.13 ***  (0.11, 0.16) |
|  | *Medium/high cost* | *Coefficient: B*  *(95% CIs)* | 0.28 ***  (0.26, 0.30) | 0.28 ***  (0.23, 0.33) |
| **p<0.05; ** p<0.01; *** p<0.001.* **^1^** Original models are those multivariable regression models presented in the main text, which adjust for region (10 regions of the United Kingdom) using dummy variable coding as a means of controlling for possible confounding by region. **^2^** Hierarchical models cluster 24,879 households into 10 regions as a means of accounting for possible clustering effects. | | | | |
